# Infants Consider the Distributor’s Intentions in Resource Allocation

**DOI:** 10.3389/fpsyg.2020.596213

**Published:** 2020-10-30

**Authors:** Karin Strid, Marek Meristo

**Affiliations:** Department of Psychology, University of Gothenburg, Gothenburg, Sweden

**Keywords:** distributive fairness, social cognition, infancy, moral development, infant development

## Abstract

Recent experimental studies suggest that preverbal infants are able to evaluate agents on the basis of their distributive actions. Here we asked whether such evaluations are based on infants’ understanding of the distributors’ intentions, or only the outcome of their actions. Ten-month-old infants observed animated movies of unequal resource allocations by distributors who attempted but failed to distribute resources equally or unequally between two individuals. We found that infants attended longer to the test event showing a third agent approaching a distributor who was unable to make an *unequal* distribution, compared to the test event where the third agent approached a distributor who was unable to make an *equal* distribution of resources. Our results suggest that infants’ ability to encode distributive actions goes beyond an analysis of the outcome of these actions, by including the intentions of the distributors whose actions lead to these outcomes.

## Introduction

In most social interactions, adult humans interpret and evaluate others’ actions based on their state of mind. The focus on mental states is crucial in making moral evaluations as we most of the time put more weight on the intention of the act than on the actual outcome (Piaget, 1932; Young et al., 2010; Killen et al., 2011; Cushman, 2015). The ability to understand others’ intentions has been shown to develop already in the first year of life (Behne et al., 2005; Southgate et al., 2008; Scott and Baillargeon, 2017), but it is as yet unclear whether infants are able to use this understanding in the context of distributive fairness. The present study investigated whether infants consider agents’ fair and unfair intentions in evaluating their unequal distribution of resources.

Experimental research has demonstrated that preverbal infants have an ability to evaluate others’ actions based on their intentions and goals. One of the earliest evidence comes from studies showing that 6- to 12-month-old infants expect agents to reach for a target object in an efficient manner (Gergely et al., 1995). After observing an agent moving around an obstacle to reach for an object, infants expected the agent to reach for the same object following a shorter path when the obstacle was removed rather than following the same longer path as previously. That is, infants seemed to understand the goal of the agent’s actions and expected the agent to behave efficiently to reach that goal. More recent studies have developed this line of research further and confirmed the earlier findings (Southgate et al., 2008; Brandone and Wellman, 2009; Scott and Baillargeon, 2013; Brandone et al., 2014).

Infants’ goal attribution skills have been additionally demonstrated in preference, helping, and imitation tasks. In one study, 5-month-olds expected an agent to reach for a preferred toy that she had reached for before in another location (Woodward, 1998) instead of reaching for another toy in the same location as before. That is, the action of the agent was interpreted as having an intention and not merely as a physical movement. This task has since then been modified in many variations, and the original findings have been replicated (Luo and Baillargeon, 2005; Spaepen and Spelke, 2007). In another study, 9-month-olds were more likely to help an adult who was unwilling to give them a toy by teasing the child with it, compared to another adult who was unable to give a toy by accidentally dropping it (Behne et al., 2005). The outcome of the adults’ actions in this experiment was the same in both conditions, and therefore, the differentiation of the infants’ responses was due to the interpretation of the adults’ intentions, not the end result of their actions. Additionally, in an imitation task (Gergely et al., 2002), infants were less likely to reproduce an adult’s head movements when her hands were occupied compared to when she was free to use her hands but did not. That is, infants understood the adult’s actions as goal directed and reproduced her actions as goal directed rather than simply imitating her physical movements (see also Schwier et al., 2006; Paulus et al., 2011).

Finally, during the last 15 years, infants’ abilities to attribute epistemic mental states to other agents such as knowledge and beliefs have been demonstrated in many European and American laboratories (Scott and Baillargeon, 2017). Implicit in all tasks included in this research, and lending further support for the studies described above, is the simpler assumption that infants are skilled in inferring others’ goals and intentions. In other words, in order to draw conclusions about an individual’s erroneous search behavior based on his/her false belief or knowledge/ignorance about the situation, infants first need to identify the goals and intentions of the depicted actions.

Findings from recent research suggest that infants are sensitive to various fairness principles during their second year of life (Geraci and Surian, 2011; Schmidt and Sommerville, 2011; Sloane et al., 2012; Sommerville et al., 2013) and possibly even earlier (Meristo et al., 2016; Ziv and Sommerville, 2017; Buyukozer Dawkins et al., 2019). In one study, Sloane et al. (2012) designed a new looking time task to examine whether 19- to 21-month-old toddlers react differently to fair and unfair distribution of resources. The infants witnessed an experimenter distributing some resources to two identical puppets. In one trial, the experimenter made an equal distribution, giving one object to each puppet, whereas in the second trial, the unequal scenario, the experimenter made an unequal distribution by giving both objects to one of the puppets. The toddlers looked reliably longer at the test scene after witnessing the unequal distribution of objects, suggesting that they expected the experimenter to distribute the objects equally.

These findings have been extended recently by demonstrating that infants not only expect fair distributions but also evaluate agents based on their distributive actions (Meristo and Surian, 2013, 2014; DesChamps et al., 2016) and prefer to interact with fair agents (Burns and Sommerville, 2014; Surian and Franchin, 2017). Further research suggests that infants’ sense of fairness might be more complex than understanding a simple concept of equality and include context-sensitive information. For instance, infants find an equal distribution unexpected when individuals differ in their work effort (Sloane et al., 2012), and they expect distributors to favor in-group members when resources are scarce (Bian et al., 2018). Finally, Surian and Margoni (2020) have demonstrated that 20-month-olds are sensitive to procedural fairness and expect help to be provided to individuals in an impartial manner. Together, these findings uncover remarkably complex abilities of reasoning about fairness in young infants.

Evaluating moral acts on the basis of intentions rather than outcome marks a developmental cornerstone in children’s moral development. This was recognized by Piaget (1932), who asked children to judge the level of naughtiness. In one story, a boy accidentally makes a small ink spot when playing with his father’s ink pot, while another boy accidentally makes a big spot when he tries to please his father by filling his empty ink pot. Up to the age of 7 years, children are likely to judge the action by its outcome, that is, the bigger the damage, the naughtier the child. These studies did not test the intentional understanding explicitly because in both scenarios, the child did not intend to do the damage on purpose. Instead, it was the action leading to the damage that was evaluated as good or bad. Using more obvious cues about intentionality, later empirical studies showed that this shift in development appears to happen in preschool years (Karniol, 1978; Nelson, 1980; Van de Vondervoort and Hamlin, 2018; Margoni and Surian, 2020).

Research using looking-time paradigm suggests that even infants are able to consider others’ mental states, such as intentions and knowledge, when interpreting social interaction (Hamlin, 2013; Meristo and Surian, 2013; Choi and Luo, 2015). In Meristo and Surian (2013), 10-month-olds were shown movies depicting equal and unequal allocation of strawberries by two distributors, the fair and the unfair. Then, one of the distributors was rewarded by a third agent. The crucial comparison here was between the aware condition, where the third agent had witnessed the distributors’ previous actions, and the unaware condition, where the third agent had been hindered to witness the distributors’ actions. Infants in the aware condition looked reliably longer at the test situation when the unfair distributor was given a reward, compared to when the fair distributor was rewarded, but no comparable difference between rewarding the two distributors was found in the unaware condition. These findings suggest that infants are able to consider others’ mental states such as knowledge/ignorance in morally relevant situations such as distributive fairness.

Hamlin and her colleagues showed in a series of studies that 8- to 10-month-olds not only evaluate others based on their prosocial and antisocial actions, but that they also prefer agents who intend to be nice and helpful, even if they fail (Hamlin, 2013; Hamlin et al., 2013). More specifically, the study showed that infants preferred to interact with an agent who tried to help to open a box but failed, over an agent who tried to hinder to open a box but failed. In another study, infants were further shown to understand both accidental help and harm (Woo et al., 2017). However, no studies, so far, have investigated infants’ reasoning about agents’ intentions in a context of distributive fairness.

Do infants consider agents’ intentions in resource allocation? Here we present three experiments that explored 10-month-old infants’ ability to detect an agent’s intentions in unequal distribution of resources. Infants watched short animations where an agent attempted but failed to distribute two strawberries to two identical individuals (experimental condition). Critically, in one trial, the distributor’s intention was to deliver strawberries equally, and in the second trial, the intention was to deliver strawberries unequally. The outcome was always an unequal distribution. We reasoned that if infants are sensitive to the distributors’ intentions, they would expect a third agent to approach the distributor with fair intentions compared to the distributor with the unfair intentions. We provide two additional conditions to examine alternative interpretations. In the inanimate–control condition, we removed the individuals who were treated unfairly by the distributor and left everything else the same, in order to rule out that infants’ reactions in the experimental condition were affected by symmetrical/asymmetrical motion in the two trials. In the affiliation–control condition, we removed the strawberries from the scene in order to examine the possibility that infants’ reactions in the experimental condition were due to the distributor’s affiliative behavior and not fairness considerations.

We decided to include 10-month-old infants because these are the youngest who have been shown to have the ability to detect the most basic and simple principle of equality in previous studies (Meristo et al., 2016; although see Buyukozer Dawkins et al., 2019, for experiments with 4-month-olds).

## Materials and Methods

### Participants

Forty-eight full-term healthy infants participated (age range = 9 months 18 days to 10 months 15 days; mean = 10 months 2 days; 24 female, 24 male). The sample size was specified on the basis of the effect size from Meristo and Surian (2013) that examined infants’ reasoning about resource distributions in the context of similar animated events and used the violation-of-expectation method and a 2 × 2 between-subject design. The condition × event effect size (η*_*p*_*^2^) in their study was 0.17. The *a priori* power analysis using G^∗^Power, based on this previous effect size, suggested that 80% power at the α level of *p* = 0.05 required a minimum number of eight participants per cell for a 3 × 2 design. Additional 16 infants were tested but excluded because they were fussy (*n* = 2), overly active (*n* = 2), inattentive (*n* = 6), because of technical problems (*n* = 2) and experimenter error (*n* = 1), or because of they had looking times that were more than 2 standard deviations (SDs) from the condition group mean (*n* = 3).

### Materials and Procedure

**FIGURE 1:**
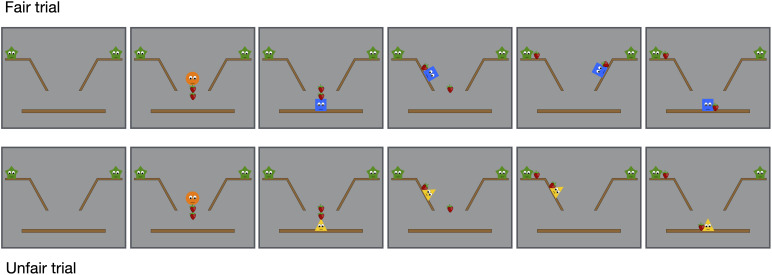
Selected frames from the distributive events of the experimental condition.

In the experimental condition, all infants were shown four distributive events in the initial phase followed by one test event in the final phase. The distributive events consisted of two fair trials and two unfair trials. The initial phase started with two green stars present on the shelf on the upper right and the upper left part of the screen (Figure 1). Then, an orange circle appeared from above with two strawberries, placing these in the middle of the screen and then leaving. Next a distributor, a yellow triangle or a blue square, arrived from left or right, and started to deliver the strawberries to the stars. In the fair trial (Supplementary Video S1), the distributor first delivered one strawberry to one of the stars and then attempted to reach the other star but failed to climb the second hill to deliver the second strawberry. In the unfair trial (Supplementary Video S2), the distributor starts with delivering the first strawberry to the first star and then attempts to climb the same hill for the second time to deliver the second strawberry to the first green star but without success. Thus, in both trials, the distributor first attempted to climb the first hill twice, each time falling back halfway, but then succeeded to reach the top of the hill on the third attempt. Then, the distributor repeatedly attempted to climb the hill for the second time, falling back three times and then giving up and failing. The video stimuli were designed to depict the idea that it requires considerable effort to deliver the strawberries (i.e., it requires several attempts), because the hills are steep and the strawberries large, and that the distributor therefore gets tired and fails the second time.

The initial phase ends with the distributor resting in the middle of the screen with a strawberry next to it. Thus, both distributors attempted but failed to deliver both strawberries to the stars, and both trials ended with an unequal distribution of strawberries where only one of the two stars had a strawberry. But in one case (the fair trial), the distributor intended to make an equal distribution, and in the second case (the unfair trial), the distributor intended to make an unequal distribution. Thus, both distributors were equally altruistic by intending to give away both strawberries. Each distributive event ended when the infant (a) looked away for more than 2 consecutive seconds or (b) looked for a maximum of 30 s.

**FIGURE 2:**
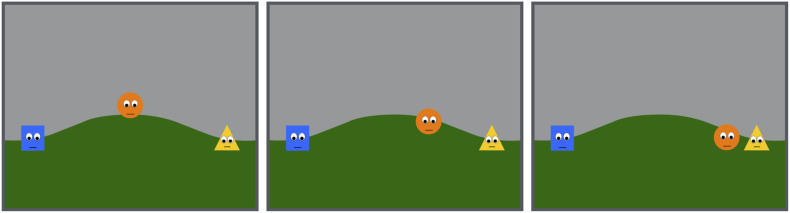
Selected frames from the test event.

In the final phase, the two distributors, the fair and the unfair distributors, rested on the right and the left side of a hill in the middle of the screen (Figure 2). The orange circle that brought in the strawberries in the initial phase was now on the top of the hill, in the middle of the two distributors. The orange circle then approached and stayed close to one of the two distributors (Supplementary Video S3). Half of the infants saw the orange circle approaching the fair distributor, and the other half saw the circle approaching the unfair distributor.

**FIGURE 3:**
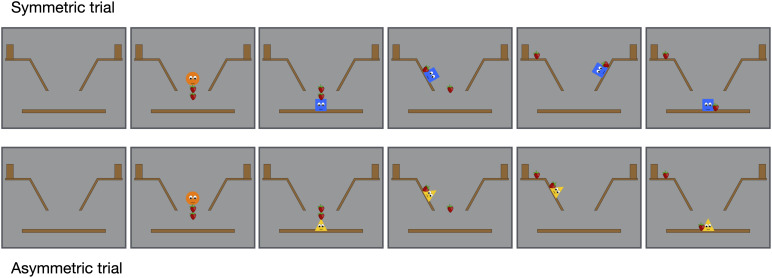
Selected frames from the displacement events of the inanimate–control condition.

To rule out the possibility that infants’ looking times reflect a non-social perceptual bias for symmetrical motions, we conducted the *inanimate–control* condition where the events were identical to those used in the experimental condition except that the animate green stars with eyes and mouth in the distributive events were replaced by inanimate brown rectangles without eyes and mouth. In the initial phase, one agent, the symmetric agent, tried but failed to displace the two strawberries symmetrically, one on the left and the other on the right-side hill. The other agent, the asymmetric agent, tried but failed to displace the strawberries asymmetrically. Thus, here the initial phase included four displacement events consisting of two symmetric trials and two asymmetric trials (Figure 3). In the final phase, the orange circle that introduced the strawberries in the initial phase approached one of the agents.

**FIGURE 4:**
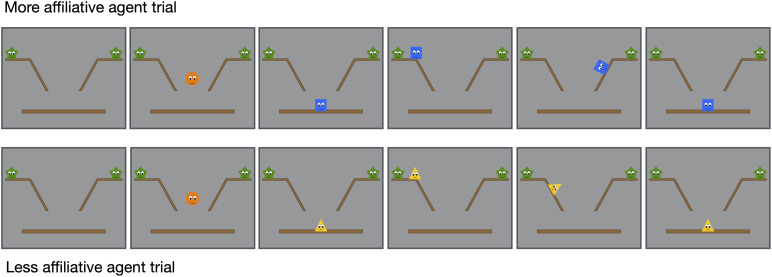
Selected frames from the more affiliative and less affiliative trials of the affiliation–control condition.

We also included a second control condition, the *affiliation–control* condition, to rule out the alternative explanation that infants evaluate more individuals who form more alliances and thus expect the third agent to approach the more affiliative individual in the test event (e.g., Powell and Spelke, 2013). That is, the fair/unable distributor in the experimental condition approaches both green stars, whereas the unfair/unable distributor approaches only one star. In the affiliation–control condition, we removed the strawberries from the distributive events in the initial phase and left everything else unchanged; that is, the initial phase included four affiliation events consisting of two more affiliative trials and two less affiliative trials (Figure 4). We hypothesize that infants would look about equally in the two test trials of the final phase (i.e., the more affiliative agent and the less affiliative agent trial) and thus confirm that the differences in the experimental condition were due to their sensitivity of the intentions of the fair and unfair distributors of the strawberries. Note that the affiliative-control condition would also help to rule out another lower-level explanation, that infants see in the initial phase the fair distributor being nearby different agents, while the unfair distributor approaching only one of the stars, and it may be less novel in the final phase for infants to see the fair/unable distributor next to a new shape (i.e., the orange circle) than to see the unfair/unable distributor next to a new shape.

Infants’ looking times were measured in the test event in all three conditions from the moment when the orange circle finished approaching one of the distributors (Figure 2), until he/she (a) looked away for at least 2 consecutive seconds after having looked for at least 2 s or (b) looked at the scene for a maximum of 60 s.

The following variables were fully counterbalanced across the participants: (1) identity of the fair/unable distributor (yellow triangle vs. blue square), (2) order of trials of the distributive events (fair–unfair–unfair–fair vs. unfair–fair–fair–unfair), (3) side of the fair distributor in the test event (left vs. right), and (4) type of test event (approaching fair distributor vs. approaching unfair), resulting in 16 different testing sessions. The side of delivery of the first strawberry in the distributive events (left vs. right) covaried with the identity of the fair distributor (i.e., blue fair distributors always delivered the first strawberry to the green star on the left). All infants who were included in the final analyses were very attentive and followed at least three trials of the distributive events.

## Results

Infants’ looking times were analyzed in a 3 × 2 analysis of variance (ANOVA) with condition (experimental, inanimate–control, or affiliation–control) as one of the between-subject factors, and test event (approach fair distributor or approach unfair distributor) as the second between-subject factor. The analysis revealed a marginally significant condition × test event interaction, *F*(2,42) = 3.21, *p* = 0.050, η*_*p*_*^2^ = 0.13, but no significant main effects.

**FIGURE 5:**
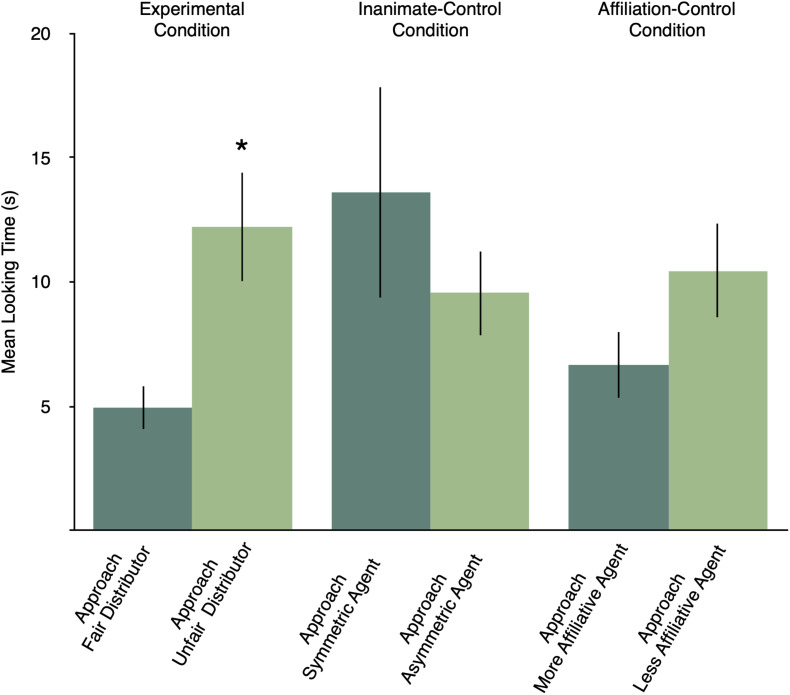
Mean looking times at the test events of experimental, inanimate–control and affiliation–control conditions. Error bars represent standard errors. *p < 0.05.

Planned contrasts showed that in the experimental condition infants looked significantly longer when the circle approached the unfair distributor (mean = 12.21, SD = 6.25), compared to when the circle approached the fair distributor (mean = 4.92, SD = 2.42), *t*(14) = 3.08, *p* = 0.013, *d* = 1.54 (Figure 5). By contrast, looking times at the two types of the test event in the inanimate–control condition did not differ (asymmetric agent approached: mean = 9.55, SD = 4.80; symmetric agent approached: mean = 13.61, SD = 11.91, *t*(14) = 0.89, *p* = 0.387, *d* = 0.45). Similarly, there were no differences in looking times in the affiliation–control condition (less affiliative agent approached: mean = 10.43, SD = 5.31; more affiliative agent approached: mean = 6.67, SD = 3.73, *t*(14) = 1.64, *p* = 0.123, *d* = 0.85).

Additional two 2 × 2 ANOVAs revealed a significant condition × test event interaction when comparing the experimental condition with the inanimate–control condition [*F*(1,28) = 4.91, *p* = 0.035, η^2^ = 0.15], but not in the comparison between the experimental and the affiliation–control conditions [*F*(1,28) = 1.14, *p* = 0.294, η^2^ = 0.04]. Therefore, any strong conclusion from a comparison of the results obtained in the experimental and the affiliation–control conditions should be interpreted with caution.

We also carried out an analysis with infants’ looking times at the still picture after each event of the initial phase (i.e., the four distributive events) as the dependent variable. The 3 × 2 ANOVA with condition (experimental, inanimate–control, or affiliation–control) as the between-subject factor, and initial phase (fair/symmetric/more-affiliative or unfair/asymmetric/less-affiliative trial) as the within-subject factor, yielded no significant interaction (*p* = 0.382) or main effects (*p*’s > 0.21). This suggests that infants attended about equally to both kinds of trials during the initial phase in all three conditions.

These results support the conclusion that infants considered the distributors’ fair or unfair intentions in a distributive situation where the outcome of the distribution was always unequal. They expected the orange circle to approach the distributor with fair intentions and looked longer when the circle approached the distributor with unfair intentions instead.

## Discussion

Do infants evaluate distributive agents on the basis of their intentions, or on the basis of the outcome of their actions? In the present experiment, we designed three conditions to test this question. In the experimental condition, we showed infants a fair distributor, who tried but was unable to make an equal distribution, and an unfair distributor, who tried but was unable to make an unequal distribution. That is, both distributors were equally generous in their donations, and the outcome of the distribution was identical. Infants’ looking times suggest that they were sensitive to the intentions of the fair and unfair distributors. In the inanimate–control condition, infants did not show any differences in their looking times when the strawberries were distributed to rectangles without eyes and a mouth, instead of individuals. In the affiliation–control condition, there were no differences in looking times between the two trials in the test phase, where the third agent approached an agent with more affiliative intentions versus an agent with less affiliative intentions. Together, these results suggest that 10-month-old infants are able to detect distributors’ intentions in a situation of resource allocation with an unequal outcome.

Findings from a couple of recent studies have suggested that infants and toddlers possess an early developing ability to detect fairness in resource allocation (Sloane et al., 2012; Sommerville et al., 2013; DesChamps et al., 2016; Meristo et al., 2016). Another line of research has proposed that infants at a very young age are also able to reason about others’ intentions (Spaepen and Spelke, 2007; Southgate et al., 2008; Woodward, 2009). The results from our current study indicate an ability in preverbal infants to combine expectations of equal distribution of resources with their skills of detecting an intended, although unfulfilled, action of an agent. Traditionally, it has been assumed that when children are asked to reason about social–moral actions, it is not until the age of 7 years that they take into account the agent’s intention of the action in their evaluation (Piaget, 1932). More recently, it has been argued that the methodology in the previous studies has consequently underestimated the children’s abilities in this regard (Hamlin, 2013; Margoni and Surian, 2016; Meristo et al., 2016). These findings support the results from other recent studies suggesting that infants’ evaluation of social–moral interactions goes beyond the mere physical and perceptual outcomes of the agents’ behavior and includes more advanced reasoning about the agents’ internal and invisible mental states such as beliefs, knowledge, and intentions (Meristo and Surian, 2013; Woo et al., 2017).

In our test scenario, the third agent (the orange circle), who brought the strawberries to the scene before the distributors appeared (the blue square and the yellow triangle), left the scene and did not witness the distributors’ actions. The orange circle was therefore ignorant about the distributors’ actions in the final phase and did not have any reason to prefer one over the other when approaching them. Therefore, infants looked longer when the circle approached the unfair distributor because they attributed their own knowledge of the events to the circle. Previous experiments have demonstrated that infants are sensitive to third agents’ epistemic mental states in the context of rewarding distributive agents when this information is explicitly provided (Meristo and Surian, 2013, Experiment 3) but attribute their own understanding to third agents when this information is left out (Meristo and Surian, 2013, Experiment 1; Meristo and Surian, 2014, Experiments 1 and 2). However, irrespective of the infants’ understanding of the third agent’s knowledge/ignorance of the preceding events, the current results support our hypothesis; that is, the infants were able to distinguish between the distributors based only on their fair and unfair intentions.

Previous research suggests that change of possession is not coded as giving by preverbal infants if it is separated into two segments without any contact between the agents (Schöppner et al., 2006). Because the third agent in our study leaves the scene before the distributors enter, its actions could be interpreted as rejection of the resources rather than giving these to the distributors. However, even if the third agent’s intentions during the initial phase might have alternative interpretations, our hypothesis about the distributors’ intentions is supported by the current results.

There are several limitations to the current study. Our findings should be interpreted with caution because the conclusions are based on small samples, and therefore, replications with larger samples are needed in order to specify the nature of this very early understanding of intentions in the context of distributive justice. Relatedly, we cannot rule out the possibility that infants’ reactions were driven by their reasoning about affiliative actions of the distributors due to the non-significant interaction between the experimental and affiliation–control condition. Finally, the present results are also limited to a very narrow context of unequal outcome of agents with fair and unfair intentions, while it could be extended to contexts where fair and unfair outcomes are caused by actions of intentional and accidental distributors (Woo et al., 2017).

A lot of previous research has demonstrated that it takes several years before children are able to verbally reason about moral intentions (Killen et al., 2011; Cushman et al., 2013; Proft and Rakoczy, 2019), yet our results suggest that a basic sense of fairness that includes reasoning about intentions is present already in preverbal infants. We suggest that the preverbal intuitive evaluations correspond to older children’s and adults’ implicit judgments, while the verbal reasoning emerging in preschoolers could be a part of our conscious and rational verbal reasoning. A recent work has argued for a developmental conceptual continuity in demonstrating that even young preschoolers were able express intent-based judgments when task demands were reduced (Margoni and Surian, 2020). Classic theories on moral development (Piaget, 1932; Kohlberg, 1981) emphasize the development of children’s explicit verbal moral reasoning and how this depends on language development, cognitive maturity, and peer interaction but do not explain the acquisition and the development of the non-verbal intuitions, which might be present very early in development.

## Data Availability Statement

All datasets presented in this study are included in the article/Supplementary Material.

## Ethics Statement

The studies involving human participants were reviewed and approved by the Regional Swedish Government Ethical Review Board. Written informed consent to participate in this study was provided by the participants’ legal guardian/next of kin.

## Author Contributions

KS and MM designed the study, prepared the experimental materials, and carried out the data collection and the statistical analyses. KS wrote the first draft of the manuscript. MM provided the revisions. Both authors contributed to the article and approved the submitted version.

## Conflict of Interest

The authors declare that the research was conducted in the absence of any commercial or financial relationships that could be construed as a potential conflict of interest.
